# Cardiovascular Properties of the Androgen-Induced PCOS Model in Rats: The Role of Oxidative Stress

**DOI:** 10.1155/2021/8862878

**Published:** 2021-08-31

**Authors:** Jovana Joksimovic Jovic, Jasmina Sretenovic, Nikola Jovic, Jovan Rudic, Vladimir Zivkovic, Ivan Srejovic, Katarina Mihajlovic, Nevena Draginic, Marijana Andjic, Milica Milinkovic, Zoran Milosavljevic, Vladimir Jakovljevic

**Affiliations:** ^1^Department of Physiology, University of Kragujevac, Faculty of Medical Sciences, Svetozara Markovica 69, 34000 Kragujevac, Serbia; ^2^Department of Gynecology and Obstetrics, University of Kragujevac, Faculty of Medical Sciences, Svetozara Markovica 69, 34000 Kragujevac, Serbia; ^3^University Clinic for Gynecology and Obstetrics “Narodni Front”, Belgrade, Serbia; ^4^Department of Pharmacy, University of Kragujevac, Faculty of Medical Sciences, Svetozara Markovica 69, 34000 Kragujevac, Serbia; ^5^Department of Histology and Embryology, University of Kragujevac, Faculty of Medical Sciences, Svetozara Markovica 69, 34000 Kragujevac, Serbia; ^6^Department of Human Pathology, I.M. Sechenov First Moscow State Medical University, Moscow, Russia

## Abstract

Polycystic ovary syndrome (PCOS) is a multifaced reproductive endocrinopathy affecting 6-20% of women of childbearing age. It was previously shown that women with PCOS have an increased risk of cardiovascular (CV) diseases. The aim of this study was to evaluate the cardiodynamic parameters of isolated rats' hearts, blood pressure levels, and histomorphological changes in the heart tissue following the androgen-induced PCOS model in rats and the role of oxidative stress in the development of these CV properties of PCOS. 21-day-old female rats (*n* = 12) were divided into control and PCOS groups. PCOS was induced by administration of testosterone enanthate (1 mg/kg BW, daily) during 35 days. During the autoregulation protocol (40-120 mmHg) on the Langendorff apparatus, ex vivo cardiodynamic parameters of retrogradely perfused hearts showed enhanced contractile function and increased lusitropic effects in the left ventricle (LV) in PCOS rats. Systolic and diastolic pressures in LV were elevated at all perfusion pressure values. Systemic arterial systolic blood pressure showed borderline elevation, while mean arterial blood pressure was significantly higher in PCOS rats. Histological evaluation of heart tissue depicted hypertrophic (8.3%) alterations in LV cardiomyocytes and increase (7.3%) in LV wall thickness. Oxidative stress parameters were altered in systemic circulation, coronary venous effluent (CVE), and heart tissue. Levels of superoxide dismutase and reduced glutathione were decreased in blood and heart tissue, while catalase activity was not altered. Degree of lipid peroxidation was increased in circulation as well as heart tissue. Increased levels of O_2_^−^ in CVE indicated the cardiotoxic effects in the rat PCOS model. The mentioned alterations of oxidative stress parameters in the blood, CVE, and heart could be recommended as potential contributors underlying the development of CV risk in PCOS women.

## 1. Introduction

Polycystic ovary syndrome (PCOS) is a multifaced reproductive endocrinopathy affecting 6-20% of women of childbearing age [1]. Differences in clinical presentations of this complex syndrome, with less or more dominant reproductive, endocrine, or metabolic features, were considered a part of the phenotype the patients expressed. The Rotterdam and Androgen Excess-PCOS Society criteria recognize 3 variants of this complex syndrome: Frank PCOS (oligomenorrhea, hyperandrogenism, and polycystic ovaries), ovulatory PCOS (hyperandrogenism, polycystic ovaries, and regular menstrual cycles), and nonpolycystic ovary PCOS (oligomenorrhea, hyperandrogenism, and normal ovarian morphology). The Rotterdam criteria also reported the fourth phenotype—normoandrogenic PCOS (oligomenorrhea, polycystic ovaries, and normal androgen levels), considered the least represented in the PCOS population [2].

Although it is well known that PCOS could affect the rate of conception, pregnancy outcome, and birth weight, it is worth mentioning that this at first sight reproductive disorder implies a high risk of CV morbidity [3]. Even singleton pregnancies in PCOS patients were frequently complicated by gestational diabetes, pregnancy-induced hypertension, and preeclampsia [4]. Young PCOS patients had a compromised CV profile and a higher risk of CV events, such as myocardial infarction [5]. Moreover, it is estimated that about 40% of women with PCOS had high blood pressure (BP) levels [6], even independently of obesity. Although obesity could be considered the mutual feature for PCOS and hypertension (HT) [7], the existence of HT in a woman with a normal body mass index potentially excludes obesity as the main contributor of hypertension development in PCOS [8]. Moreover, it is believed that young PCOS women manifest a prehypertensive state [9] and have obesity-independent increased intima-media thickness, which directly correlates with serum androgen levels [10].

Animal and human studies showed similar results concerning the CV and metabolic outcomes in PCOS. One of the most common animal models for PCOS induction represents the chronic androgen administration to prepubertal rats [11]. Although it is well known that there is no perfect animal model which mimics all human PCOS features, these models could be useful for investigating different PCOS aspects. In this sense, the androgen-induced PCOS model could be appropriate to examine both ovarian morphology and metabolic aspects of PCOS. Regarding the PCOS rat model and CV features, it is known that rats with PCOS showed increased sympathetic activity and HT [12], as well as impaired NO synthase and Na/K-ATP-ase activity in the heart tissue [13].

Although the exact etiopathogenetic mechanisms of PCOS are not fully revealed yet, oxidative stress and low-grade inflammation were proposed as underlining mechanisms in PCOS models [14]. These alterations in oxidative and inflammatory parameters showed a strong correlation with the levels of circulating androgens [15]. Literature data revealed the controversial roles of testosterone regarding oxidative stress. There are studies showing beneficial, antioxidant effects in the rat heart following testosterone administration in lower doses [16]. On the contrary, prooxidative effects of testosterone and its derivatives on CV [17], endocrine [18], and behavioral [19, 20] properties were proven in our previous papers, dealing with the administration of supraphysiological doses in male rats. Investigations with similar protocols in female rodents have not been represented in literature so far, except androgen administration to female rats to mimic PCOS characteristics [13]. The data concerning PCOS models demonstrated increased oxidative stress with impairment of antioxidant protection in different tissues, such as liver, muscle, and periovarial fat tissue [21].

However, the CV response in the androgen-induced PCOS model in the isolated rat heart has not been investigated yet. Moreover, oxidative stress parameters in the heart tissue as well as in coronary venous effluent (CVE) were not determined in the rat PCOS model. Therefore, the purpose of this study was to evaluate the CV features of the androgen-induced PCOS model in rats, measuring cardiodynamic parameters in the isolated rat heart, and the role of oxidative stress in the development of these PCOS properties.

## 2. Materials and Methods

### 2.1. Ethics Statement

All research procedures were carried out in strict accordance with the European Directive for the welfare of laboratory animals (No. 86/609/EEC) and principles of Good Laboratory Practice and ARRIVE guidelines. Ethical permission was approved by the Ethical Committee of the Faculty of Medical Sciences, University of Kragujevac, Serbia. Efforts were made to minimize the number of animals used and their suffering.

### 2.2. Animals

Three-week-old female Wistar albino rats (*n* = 12), weighing 50–60 g, were obtained from the Military Medical Academy, Belgrade, Serbia. Rats were kept under controlled environmental conditions (23 ± 1°C, 12 : 12 h light/dark cycle—lights on 08:00 h), with *ad libitum* access to food and water.

### 2.3. Experimental Design

The rats were randomly divided into two groups (6 animals per group): control (CTRL) and PCOS group (PCOS). PCOS was induced as a daily subcutaneous injection of testosterone enanthate (TE, Galenika a.d., Serbia), dissolved in sesame oil (1 mg/100 g of body weight (BW)) for 5 weeks [22, 23]. Animals from the CTRL group received 0.1 ml sesame oil for 5 weeks. During the protocol, BW was measured daily to calculate drug doses. The injections were administered daily in the same interval (11:00-11:30 h) in order to minimize the effect of manipulation on animal welfare. Twenty-four hours after the last TE injection, animals were anesthetized by intraperitoneal application of ketamine (10 mg/kg) and xylazine (5 mg/kg) and sacrificed by decapitation. The trunk blood samples were collected, while the hearts were isolated from the chest cavity and prepared for the Langendorff apparatus. The obtained serum, plasma, and erythrocyte lysate samples were stored at -20°C for further analysis. Ovaries were harvested for further pathohistological analysis. All animals were sacrificed at the same estrus phase (diestrus) to eliminate the cycle phase impact on analyzed parameters.

### 2.4. Assessment of the Estrous Cycle

During the last two weeks of the protocol, the estrus cycle was monitored daily by cytological examination of vaginal smears. Briefly, every morning at 9:00 h, before treatment, vaginal lavage was performed with a dropper filled with a small volume of distilled water. The lavage was placed on a glass slide and stained with hematoxylin and analyzed by using a light microscope. Estrus cycle phases were identified by predomination of specific cells as follows: proestrus—round, nucleated cells; estrus—cornified squamous cells; metaestrus—cornified squamous cells and leucocytes; and diestrus—nucleated epithelial cell and predominance of leucocytes [24].

### 2.5. Evaluation of Arterial BP Levels

A day before sacrificing, the noninvasive method for the evaluation of systolic and diastolic arterial BP, as well as heart rate in rats, was performed using the BP system (Rat Tail Cuff Method Blood pressure Systems (MRBP-R), IITC Life Science Inc., Los Angeles, CA, USA) [25]. Mean arterial BP was determined by the following formula: (MAP) = [systolic blood pressure + (2 × diastolic blood pressure)]/3. Pulse pressure (PP) was calculated as the difference between systolic and diastolic blood pressures.

### 2.6. Isolated Rat Heart Preparation

After 5 weeks of treatment, in the diestrus cycle phase, all rats were sacrificed. After short ketamine/xylazine narcosis, emergency thoracotomy was performed, and hearts of rats were immersed in ice-cold saline and attached to the Langendorff apparatus via an aortic cannula. The hearts were retrogradely perfused according to the Langendorff technique, and gradually increased perfusion pressures (40 cm H_2_O–120 cm H_2_O) were applied. Krebs-Henseleit solution (NaCl 118 mM, KCl 4.7 mM, CaCl_2_ × 2H_2_O 2.5 mM, MgSO_4_ × 7 H_2_O 1.7 mM, NaHCO_3_ 25 mM, KH_2_PO_4_ 1.2 mM, and glucose 5.5 mM, equilibrated with 95% O_2_/5% CO_2_ and warmed to 37°C (pH 7.4)) served as the fluid for heart perfusion. Immediately after the establishment of automatic operation, a latex balloon was inserted into the left chamber, after the incision of the left atrium adjacent to a severed mitral valve. The balloon (large enough so that pressure was not generated over the LV volume used in the experiment) was filled with bubble-free saline and connected to a pressure sensor (transducer BS4 73-0184, Experimentria Ltd., Hungary) for continuous recording of the myocardial function parameters.

#### 2.6.1. Physiological Assay and Experimental Protocol

After heart perfusion was established, a 30 min period was given for the heart to stabilize. After the equilibration period (70 cm H_2_O), coronary perfusion pressure (CPP) was lowered to 40 cm H_2_O and then gradually increased to 60 cm H_2_O, 80 cm H_2_O, 100 cm H_2_O, and finally 120 cm H_2_O. The coronary flow (CF) was considered to be stable at each value of perfusion pressure when three repeated values of CF were the same. Using the sensor placed in the LV, the following parameters of myocardial function were registered: maximum rate of left ventricular pressure development (dp/dt max), minimum rate of left ventricular pressure development (dp/dt min), systolic left ventricular pressure (SLVP), diastolic left ventricular pressure (DLVP), and heart rate (HR). CF was measured flowmetrically during the protocol.

### 2.7. Heart Tissue Homogenization

After accomplishing ex vivo experiments, all hearts were measured and resected and LVs were used for further analysis. One-half of the LV section was used for histological examination while the other half was used for biochemical analysis. 0.5 g of LV tissue was homogenized in 10 volumes of ice-cold phosphate-buffered saline (pH 7.4), and the homogenate was centrifuged at 1200 × *g* at 4°C for 20 minutes. The supernatants were stored at -70°C until performing the analyses [26].

### 2.8. Biochemical Analysis

#### 2.8.1. Hormonal Assays

Serum samples were assessed for the determination of sex hormone levels: total testosterone (T), progesterone (P4), and estradiol (E2) levels [20]. T, P4, and E2 levels were determined by using the Elecsys 2010 analyzer using the method of the electrochemiluminescence immunoassay (ECLIA). Standard commercial kits (Elecsys Testosterone II, Progesterone II, and Estradiol III Roche Diagnostics, Mannheim, Germany) were used, and the T and P4 levels were expressed in ng/ml and E2 was expressed in pg/ml. The sensitivities of the assays for T, P4, and E2 were 0.025 ng/ml, 0.03 ng/ml, and 5 pg/ml, respectively. Inter- and intra-assay coefficients of variance for T, P4, and E2 were 3.8%, 3%, and 2.2% and 5%, 5%, and 3.9%, respectively.

#### 2.8.2. Oxidative Stress Parameters

In plasma samples, the following parameters were measured: index of lipid peroxidation (expressed as thiobarbituric acid reactive substances (TBARS)), level of nitrites (NO_2_^−^), hydrogen peroxide (H_2_O_2_), and superoxide anion radical (O_2_^−^). The activities of enzymatic (superoxide dismutase and catalase) and nonenzymatic (GSH) antioxidant systems were determined from the lysed erythrocyte suspension. Lysates were prepared after separation of plasma, by washing isolated erythrocytes 3 times in 3 volumes of ice-cold 0.9% NaCl. Hemolysates containing about 50 g Hb/l [27] were used for the determination of antioxidant enzymes and reduced glutathione levels. During the ex vivo heart perfusion, after the stabilization period, samples of CVE were collected at every perfusion pressure, to determine the following oxidative stress parameters: TBARS, H_2_O_2_, O_2_^−^, and NO_2_. The activity of TBARS, SOD, CAT, and GSH levels was also measured in heart tissue homogenates.

*(1) TBARS Determination*. The degree of lipid peroxidation in the plasma samples was estimated by measuring TBARS. The procedure was done by mixing 0.8 ml of sample (plasma, venous effluent, or heart tissue homogenate) with 0.4 ml trichloroacetic acid. After 15 minutes on ice and centrifugation at 6000 rpm, the supernatant was collected. 1% thiobarbituric acid in 0.05 NaOH was incubated with the supernatant at 100°C for 15 min. Spectrophotometric determination was performed at 530 nm. Distilled water solution served as a blank probe for plasma and homogenate samples, while Krebs-Henseleit solution was used for effluent [28].

*(2) Determination of NO_2_^−^*. Rapid decomposition of NO, forming stable metabolite nitrite/nitrate products, was used in the Griess reaction for the detection of nitrate and NO_2_^–^ levels: NO_2_^–^ was determined as an index of NO production with the Griess reagent [29]. 0.1 ml 3 N perchloride acid, 0.4 ml 20 mM ethylenediaminetetraacetic acid, and 0.2 ml sample were put on ice for 15 min and then centrifuged for 15 min at 6000 rpm. After pouring off the supernatant, 220 *μ*l K_2_CO_3_ was added. NO_2_^–^ was measured at 550 nm wavelength, and distilled water was used as a blank probe.

Determination of nitrites in CVE was performed as follows: 0.5 ml of the perfusate was precipitated with 200 *μ*l of 30% sulfosalicylic acid, mixed for 30 min, and centrifuged at 3000 × *g*. Equal volumes of the supernatant and Griess reagent were mixed and stabilized for 10 min in the dark, and then, the sample was measured spectrophotometrically at a wavelength of 543 nm. Nitrite concentrations were determined using sodium nitrite as the standard.

*(3) Determination of H_2_O_2_*. The measurement of hydrogen peroxide (H_2_O_2_) is based on the oxidation of phenol red by hydrogen peroxide, in a reaction catalyzed by horseradish peroxidase (HRPO). 200 *μ*l of plasma sample was precipitated with 800 *μ*l of freshly prepared phenol red solution, followed by the addition of 10 *μ*l of (1 : 20) HRPO. The level of H_2_O_2_ was measured at 610 nm, and while distilled water was used as a blank probe for plasma samples, the Krebs-Henseleit solution was used as the blank probe for effluent samples [30].

*(4) Determination of O_2_^−^*. The concentration of the superoxide anion radical (O_2_^−^) was measured after the reaction of nitro blue tetrazolium in TRIS buffer with the plasma and CVE samples. The determination was performed at 530 nm wavelength. Distilled water solution served as a blank probe for plasma samples while the Krebs-Henseleit solution was used for effluent [31].

*(5) Determination of CAT*. 50 *μ*l CAT buffer, 100 *μ*l sample, and 1 ml 10 mM H_2_O_2_ were used for CAT determination. Detection was performed at 360 nm. Distilled water was used as a blank probe, and the amount of CAT was expressed as U/g tissue or U/g Hb × 10^3^ [32, 33].

*(6) Determination of SOD*. SOD activity was determined by the epinephrine method of Misra and Fridovich [34]. 100 *μ*l sample and 1 ml carbonate buffer were mixed, and then, 100 *μ*l epinephrine was added. Detection was performed at 470 nm, and the amount of SOD was expressed as U/g tissue or U/g Hb × 10^3^.

*(7) Determination of GSH*. The level of reduced glutathione (GSH) was determined spectrophotometrically, and it is based on GSH oxidation via 5,5-dithiobis-6,2-nitrobenzoic acid. The GSH extract was obtained by combining 0.1 ml of 0.1% EDTA, 400 *μ*l plasma, and 750 *μ*l precipitation solution (containing 1.67 g metaphosphoric acid, 0.2 g EDTA, and 30 g NaCl) and filled with distilled water until 100 ml. After mixing in the vortex machine and extraction on cold ice (15 min), it was centrifuged at 4000 rpm (10 min). Distilled water was used as a blank probe. Measuring was performed at 420 nm [35].

### 2.9. Histology Assessment

#### 2.9.1. Ovarian Histology

After sacrificing the rats, the left ovary was excised, cleaned, and measured. The ovary was fixed in 10% neutral buffered formalin solution and processed for light microscopic analysis. We had no intention to evaluate folliculogenesis counting the different types of follicles, but rather to confirm ovulation indirectly by the presence of corpora lutea and detect various stages of follicular development. The 4 *μ*m thin tissue sections were stained with H/E, and the central section was analyzed at x40 magnification to estimate ovarian histomorphology, using Olympus BX-51, Olympus Europa GmbH, Germany.

#### 2.9.2. Histological and Morphometric Analysis of LV

The half section of LV was fixed in 4% neutral formalin and subsequently processed for light microscopic analysis. The 5 *μ*m thin sections were stained with the standard H/E method. The following morphometric parameters of cardiomyocytes were estimated: cross-sectional area and longitudinal section diameter of cardiomyocytes and LV wall thickness. The morphometric analysis was done at x400 magnification according to our previous methodology [36], using calibrated AxioVision software (Zeiss, USA).

### 2.10. Statistical Analysis

Values were presented as the mean ± standard error. Prior to statistical analysis, all data were checked for normality, and depending on distribution, the data were evaluated using the *t*-test or Mann-Whitney *U* test. These analyses were carried out using the SPSS statistical program version 22.0. The *p* value below 0.05 was considered to be statistically significant.

## 3. Results

### 3.1. PCOS Characteristics: Effect of TE Administration on the Estrus Cycle, BW, Ovarian Histomorphology, and Hormonal Analysis

**Figure 1:**
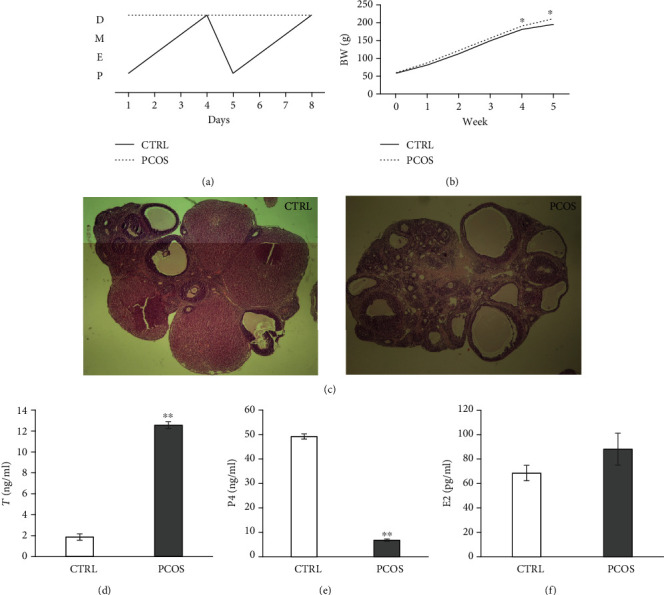
Characteristics of the PCOS rat model. (a) Estrus cycle: P—proestrus, E—estrus, M—metaestrus, and D—diestrus. (b) Body weight measurements at the beginning and during 5 weeks of TE administration. (c) Histomorphological evaluation of the central ovarian section: left—the CTRL group showed visible corpora lutea and follicles in various developmental stages; right—the PCOS group showed multiple cystic follicles, no visible corpora lutea, and many atretic follicles. (d) Serum testosterone (T) concentrations. (e) Serum progesterone (P4) concentrations. (f) Serum estradiol (E2) concentrations. Data are presented as means ± SEM. ^∗^Statistical significance at the level of *p* < 0.05 between the CTRL and PCOS groups. ^∗∗^Statistical significance at the level of *p* < 0.01 between the CTRL and PCOS groups (*n* = 6 rats per group).

Figure 1 shows the parameters observed for PCOS establishment in rats. In the last 8 days of the 5-week protocol, the estrus cycle was of normal duration (4-5 days) in the CTRL group, while at the same time, PCOS rats displayed persistent diestrus (Figure 1(a)). From the beginning of the protocol, the BW (Figure 1(b)) was similar in both groups, but a statistically significant increase in BW (*p* < 0.05) appeared in the 4^th^ and 5^th^ week in PCOS rats compared to CTRL. Normal ovarian appearance with follicles in different development stages and visible corpora lutea were observed in the CTRL group, while PCOS rats showed typical PCOS appearance: cystic structure and many atretic and preantral follicles, with no visible corpora lutea (Figure 1(c)). The hormonal status of rats is shown in Figures 1(d)–1(f). PCOS rats showed a significant increase in T levels (*p* < 0.01) after TE administration, as shown in Figure 1(d). A significant decrease in the P4 level was observed in the serum of PCOS rats (*p* < 0.01), as shown in Figure 1(e), while E2 was similar in both groups (Figure 1(f)).

### 3.2. Effects of PCOS Induction on Cardiodynamic Parameters and Coronary Flow of the Isolated Rat Heart

**Figure 2:**
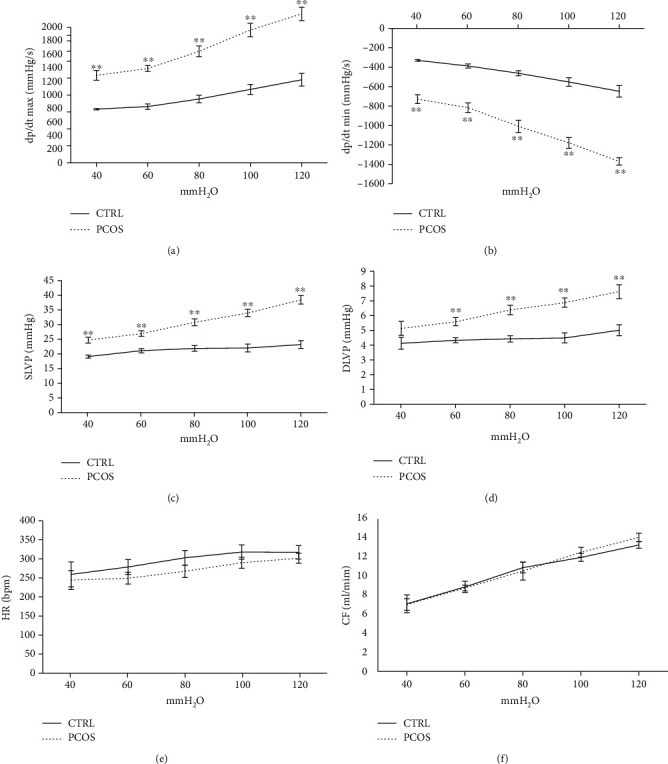
Effects of the androgen-induced PCOS model on ex vivo cardiac function. (a) Comparison between groups as to the value of dp/dt max, (b) comparison between groups as to the value of dp/dt min, (c) comparison between groups as to the value of SLVP, (d) comparison between groups as to the value of DLVP, (e) comparison between groups as to the value of heart rate, and (f) comparison between groups as to the value of coronary flow. Data are presented as means ± SEM. ^∗∗^Statistical significance at the level of *p* < 0.01 between the CTRL and PCOS groups (*n* = 6 rats per group).

The cardiodynamic parameters were altered in the PCOS group relative to CRTL, as shown in Figure 2. In PCOS rats, the maximum rate of LV pressure development (dp/dt max) was significantly increased while the minimum rate of LV pressure development (dp/dt min) was decreased (*p* < 0.01) during pressure changing protocols compared to CTRL (Figures 2(a) and 2(b)). Also, SLVP was significantly (*p* < 0.01) elevated while DLVP (except at 40 mmHg) showed significantly reduced values (*p* < 0.01) in the PCOS group (Figures 2(c) and 2(d)). However, HR and CF were not altered following the applied chronic TE protocol (Figures 2(e) and 2(f)), although registered HR was at lower values at all observed pressure values (n.s), as shown in Figure 2(e).

### 3.3. Effects of PCOS Induction on the Final BW and Heart Weight

The final BW, as well as heart weight (HW), was significantly higher in PCOS rats compared to CTRL. Relative weights of hearts, expressed as HW/BW, were significantly higher in the PCOS group, as presented in Table 1.

### 3.4. Effects of PCOS Induction on Blood Pressure Levels

**Figure 3:**
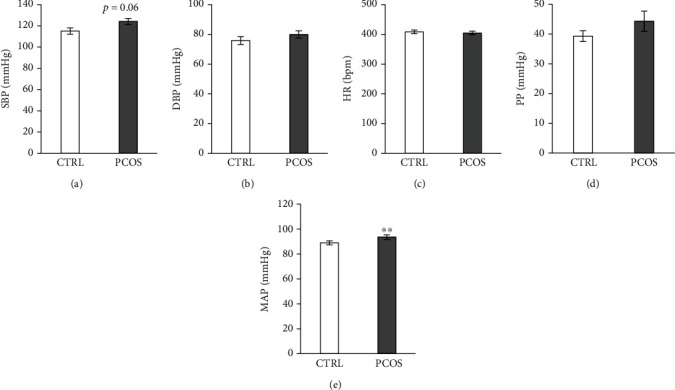
Effects of the androgen-induced PCOS model on blood pressure. (a) Systolic blood pressure (SBP), (b) diastolic blood pressure (DBP), (c) heart rate (HR), (d) pulse pressure (PP), and (e) mean arterial blood pressure (MAP). Data are presented as means ± SEM. ^∗∗^Statistical significance at the level of *p* < 0.01 between the CTRL and PCOS (*n* = 6 rats per group).

Figure 3(a) shows increased levels of systolic blood pressure (SBP) in PCOS rats compared to CTRL with borderline significance (*p* = 0.06). On the other hand, alterations in diastolic blood pressure (DBP), as well as HR and PP, were not statistically significant between the two groups. However, the observed increase in MAP in the PCOS group was statistically significant compared to that in CTRL (*p* < 0.01), as shown in Figure 3(e).

### 3.5. Effects of PCOS Induction on Cardiac Oxidative Status

**Figure 4:**
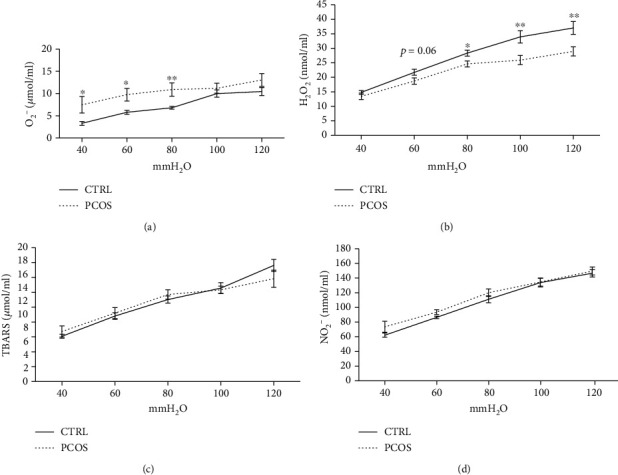
Effects of the androgen-induced PCOS model on oxidative stress parameters in coronary venous effluent. (a) Levels of O_2_^−^, (b) levels of H_2_O_2_, (c) levels of TBARS, and (d) levels of NO_2_^−^. Data are presented as means ± SEM. ^∗^Statistical significance at the level of *p* < 0.05 between the CTRL and PCOS. ^∗∗^Statistical significance at the level of *p* < 0.01 between the CTRL and PCOS (*n* = 6 rats per group).

The estimated parameters of cardiac oxidative status (Figure 4) were measured in the CVE. The values of O_2_^−^ were significantly higher in PCOS rats at lower pressure values (*p* < 0.05, *p* < 0.05, and *p* < 0.01 at 40, 60, and 80 mmHg, respectively) compared to CTRL (Figure 4(a)). On the other hand, H_2_O_2_ values were significantly lower at higher pressure levels (*p* < 0.05, *p* < 0.01, and *p* < 0.01 at 80, 100, and 120 mmHg, respectively) in PCOS rats, as shown in Figure 4(b). Also, borderline significance (*p* < 0.06) was observed in the PCOS group as a decreased concentration of H_2_O_2_ compared to the CRTL group at 60 mmHg (Figure 4(b)). The index of lipid peroxidation, as well as nitrite levels, was similar in both groups at all registered perfusion pressure values (Figures 4(c) and 4(d)).

### 3.6. Systemic Redox Status

**Figure 5:**
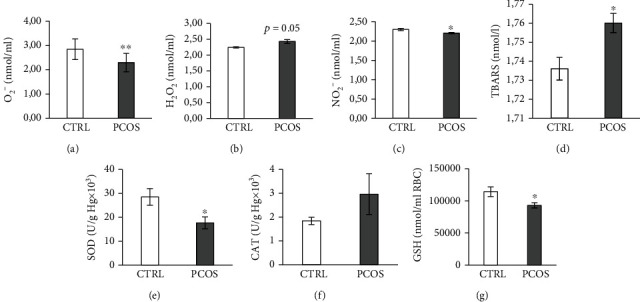
Effects of the androgen-induced PCOS model on systemic oxidative stress parameters measured in plasma. (a) Levels of O_2_^−^, (b) levels of H_2_O_2_, (c) levels of NO_2_^−^, (d) levels of TBARS, (e) levels of SOD, (f) levels of CAT, and (g) levels of GSH. Data are presented as means ± SEM. ^∗^Statistical significance at the level of *p* < 0.05 between the CTRL and PCOS. ^∗∗^Statistical significance at the level of *p* < 0.01 between the CTRL and PCOS (*n* = 6 rats per group).

As shown in Figure 5, the systemic redox state was estimated in plasma and erythrocyte lysate. The PCOS group showed significantly lower levels of O_2_^−^ (*p* < 0.01) and nitrites (*p* < 0.05) compared to CRTL (Figures 5(a) and 5(c)). However, H_2_O_2_ values were not altered (*p* = 0.05), with a tendency to increase (Figure 5(b)), while the index of lipid peroxidation was significantly increased in the plasma of PCOS rats (Figure 5(d), *p* < 0.05). The levels of SOD were significantly lowered (*p* < 0.05) in PCOS rats (Figure 5(e)), while levels of CAT were not altered (Figure 5(f)). Reduced glutathione concentration was significantly decreased in PCOS rats (*p* < 0.05), as shown in Figure 5(g).

### 3.7. Effects of PCOS Induction on Oxidative Status of LV Tissue

**Figure 6:**
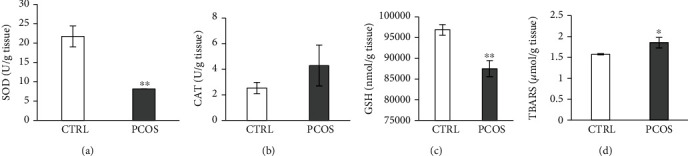
Effects of the androgen-induced PCOS model on oxidative stress parameters in LV. (a) Levels of SOD, (b) levels of CAT, (c) levels of GSH, and (d) levels of TBARS. Data are presented as means ± SEM. ^∗^Statistical significance at the level of *p* < 0.05 between the CTRL and PCOS. ^∗∗^Statistical significance at the level of *p* < 0.01 between the CTRL and PCOS (*n* = 6 rats per group).

Figure 6 shows oxidative stress parameters in the LV homogenate. SOD activity in LV tissue was lowered in PCOS rats (*p* < 0.01, Figure 6(a)), as well as GSH levels (*p* < 0.01, Figure 6(c)). CAT activity in the heart was not altered following the PCOS induction protocol (Figure 6(b)), while TBARS was significantly increased compared to that of CTRL (*p* < 0.05, Figure 6(d)).

### 3.8. Effects of Androgen-Induced PCOS on Morphometric Parameters of LV Tissue

**Figure 7:**
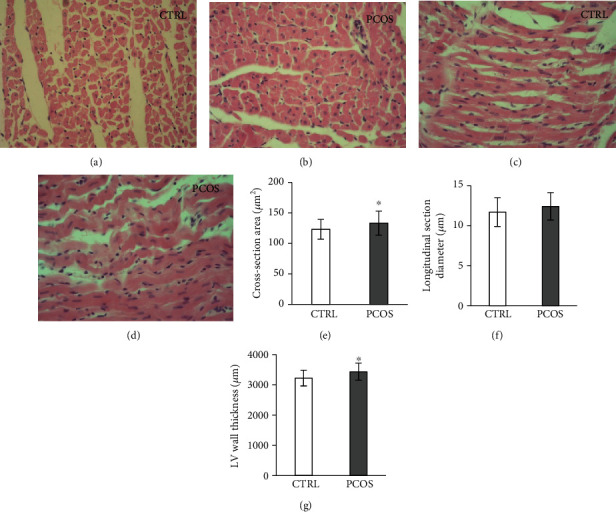
Representative photomicrographs of H/E staining of LV tissue (a–d): (a) cross LV section of CTRL, (b) cross LV section of PCOS, (c) longitudinal LV section of CTRL, and (d) longitudinal LV section of PCOS. Morphometrical parameters of LV (e–g): (e) cross-sectional area of cardiomyocytes (*μ*m^2^), (f) longitudinal section diameter (*μ*m), and (g) LV wall thickness (*μ*m). Data are presented as means ± SEM. ^∗^Statistical significance at the level of *p* < 0.05 between the CTRL and PCOS. ^∗∗^Statistical significance at the level of *p* < 0.01 between the CTRL and PCOS (*n* = 6 rats per group).

H/E-stained sections of LV tissue showed normal cardiomyocytes with no identified morphological changes in the CTRL group at both cross and longitudinal sections (Figures 7(a) and 7(c)). However, the PCOS group showed hypertrophy of cardiomyocytes with narrowed intercellular spaces (Figure 7(b)), while longitudinal sections depicted hypertrophic alterations with some wavy muscle fibers (Figure 7(d)). The cross-sectional area of cardiomyocytes in the PCOS group was significantly (*p* < 0.05) increased by 8.3% relative to the CTRL group (Figure 7(e)). Moreover, the longitudinal section diameter was slightly increased after PCOS induction in rats, but not significantly (Figure 7(f)). The LV wall thickness was significantly increased in PCOS rats (increase of 7.3%, *p* < 0.05, Figure 7(g)).

## 4. Discussion

The present study was focused on the CV effects of chronic androgen administration in female rats in order to accomplish and evaluate the hyperandrogenic PCOS model, with a special emphasis on oxidative stress. The results of our study strongly indicate that hyperandrogenism, as a common PCOS feature, can increase inotropic and lusitropic properties of LV, as well as systolic and diastolic LV pressure in the rat model. Also, PCOS rats express a tendency to increase systemic BP levels and express increased MAP. The unbalanced redox state in circulation as well as CVE and heart tissue in hyperandrogenic PCOS rats could be responsible for observed cardiac and vascular alterations. Taken together, remarkably lower activities of SOD and GSH in blood and LV tissue increased lipid peroxidation in the blood and heart; also, increased oxidative damage in CVE could be related to altered CV properties of PCOS rats. Potentially, the lower level of nitrites in PCOS rats' plasma could be responsible for the aforementioned tendency for BP increase.

Despite numerous preclinical investigations regarding PCOS, the perfect animal model has not been established yet. However, the androgen administration in a specific time period (peripubertal) was commonly used to achieve PCOS characteristics in rats [37, 38]. These similarities do not mimic a complex syndrome such as PCOS but could help researchers to have a better insight into specific characteristics of PCOS and improve the possibility for revealing mechanisms of pathogenesis and different treatment protocols in animal models. In this study, we used a protocol which was suggested by other authors for PCOS induction in mice [23] and rats [39]. The described hyperandrogenic model resulted in increased body weight, cessation of the estrus cycle, and cystic ovarian appearance in PCOS rats, as registered in our study, confirming PCOS induction. Hormonal perturbances after 5 weeks of TE administration were registered as higher testosterone, lower progesterone, and (not significantly) elevated estradiol levels in the PCOS group compared to CTRL. Although nonaromatizable androgens, such as dihydrotestosterone (DHT), were more frequently used in PCOS animal models, our study clearly indicates that estradiol levels did not change remarkably after 5 weeks in female rats. The state of hyperandrogenism and lowered progesterone could be responsible for the ovulation absence (absence of the corpus luteum) and cystic ovarian appearance (Figure 1(c)). The hormonal analysis of our study is in line with similar reports [23, 40, 41], and insignificant elevation of estradiol, in addition to body and ovary weight changes, was confirmed by others after T administration in female rats [42]. However, other authors demonstrated different hormonal profiles in various animal PCOS models [11]. Those discrepancies could be attributed to different strains, treatments, and ages of animals. One of the limitations of our study was the technical impossibility to measure aromatase (CYP19) levels, which could explain the alterations in registered hormone values. However, the finding of markedly higher T level, in addition to insignificant elevation of E2 level, suggests suspicion that the metabolic pathway was predominantly shifted towards DHT formation.

Although the role of estrogen in cardioprotection was well established, the role of androgen in females remains controversial. There are conflicting results concerning the effects of T on cardiac function. Lower doses could exert beneficial effects through antioxidative, anti-inflammatory, antiarrhythmic, and vasodilatory properties and reduction of myocardial infarct size they expressed [43]. On the other hand, many deleterious effects of testosterone on the CV system were proven: sudden cardiac death, HT, microthrombosis, and accumulation of extracellular collagen [44]. Moreover, administration of supraphysiological doses of anabolic androgenic steroids, such as ND, promotes oxidative damage and disturbed redox balance [45]. There is evidence that both low and high doses of testosterone in chronic protocols suppressed ventricular remodeling and improved left ventricular function, reduced apoptosis, and prevented mortality in a congestive heart failure rat model [46]. However, supraphysiological doses, applied in our investigation, rather depicted worsening of CV function.

During ex vivo cardiac examination using retrograde perfusion of the PCOS rat's hearts on the Langendorff apparatus, increased contractility during the whole pressure raising protocol was registered. Hyperandrogenic PCOS rats show elevation in maximum and decrease in minimum rates of LV pressure development during isovolumetric contraction. On the other hand, both systolic and diastolic pressures developed in LV, during the same protocol, were above the control values. These findings suggest that high androgen levels in chronically pretreated female rats could be the responsible factors for cardiodynamic parameter alterations in the isolated rat heart. These observations could indicate the promotion of the inotropic state in the rat heart after chronic T treatment. There is a lack of literature data concerning the effects of the mentioned treatment on cardiodynamic parameters in female rodents to compare the data to males. Golden and coworkers evaluated the effect of T on isolated ventricular myocytes, and the main observation was that T could alter calcium homeostasis via regulation of the expression of calcium regulatory proteins in the heart [47]. A recent study examined the effects of physiological T in normal and castrated male rats and concluded that T improved heart contractility in response to *α*1- and *β*1-adrenoceptor stimulation [48]. In this study, authors administered T in lower doses and, in the presence of *α*1- and *β*1-adrenoceptor agonists, estimated the velocity of contraction and relaxation as well as ventricle pressure development. Maximum and minimum rates of left ventricular pressure development increased in T-treated rats in the presence of *α*1- and *β*1-adrenoceptor agonists, which were expressed as increased contractility and speedier relaxation of the heart muscle. These crosstalks between androgens and adrenoceptors were associated with increased Ca^2+^ release via the ryanodine receptor and faster Ca^2+^ removal out of the cytosol via SERCA and Na/Ca exchanger. Although in the mentioned study, physiological concentrations of T alone did not alter cardiodynamic parameters, in our study, the enhanced inotropic properties of female rat hearts were accompanied by higher T levels. In addition, the elevated SLVP and DLVP were observed in our study during the raising perfusion pressure protocol. Due to a lack of literature data, these findings could be compared only with the acute administration protocol in isolated rat heart experiments. The acute administration of nandrolone decanoate (ND) decreased contractile function at higher CPP values, which was accompanied by lower SLVP [49]. Very recent data concerning the PCOS rat model after the ischemia-reperfusion protocol in the isolated rat heart, in basal condition, did not find changes in hemodynamic parameters, although the DLVP tended to increase [50].

The increased (8.3%) cross-sectional area of cardiomyocytes was registered in PCOS rats, as well as LV thickness (7.3%), while the longitudinal section diameter did not show significant elevation after TE treatment. These histomorphological changes were in line with the enhanced inotropic state in treated rats. It is long known that androgens act directly on cardiomyocytes inducing their hypertrophy via an AR-specific mechanism [51]. Studies concerning the effects of androgen administration on heart tissue mostly relied on chronic protocols that mimic anabolic androgenic steroid abuse in humans. In that sense, administration of synthetic derivatives of testosterone, such as ND, showed detrimental effects on CV function which were accompanied by cardiac hypertrophy and proliferation of coronary smooth muscle cells [17]. The underlying mechanism of the observed alterations was described as oxidative disturbance with a focus on enhanced lipid peroxidation, homocysteine levels, and 8-OHdG in heart tissue. The literature data supports the claim that supraphysiological T doses induce maladaptive cardiac hypertrophy [52] and that molecular mechanisms responsible for the deterioration of heart function include mTOR and ERK1/2 pathways, at least in part, which were also connected to oxidative stress production. Oxidative damage registered in our study showed decreased antioxidant capacity in heart tissue as well as systemic circulation. In the study by Nikolic et al., the similar protocol of ND administration resulted in a prooxidative state in systemic circulation [45]. As in our study, pronounced lipid peroxidation, as well as decreased enzymatic and nonenzymatic antioxidant defense system, was described in an adolescent male rat study, accompanied by high T levels [53]. Similar results were obtained after ND treatment by others who proved impaired cellular redox imbalance by decreasing antioxidant protective mechanisms [54]. The authors showed reduced activity of SOD and reduced thiols, with no change in CAT activity in heart homogenates, as observed in our study. Moreover, their results showed a positive correlation between total testosterone levels, and MDA in LV tissue was observed in a study with high testosterone doses during 6 weeks [53]. In our research study, there was also a significant positive correlation between TBARS in LV and levels of testosterone in circulation (*R* = 0.57, *p* < 0.05, data are not represented in Results). Since it is known that elevated testosterone levels enhance the activity of hormone-sensitive lipase and promote lipolysis in cardiomyocytes, keeping long long-chain fatty acids free for ATP synthesis and following augmentation of reactive oxygen species (ROS) production also [55], it could be concluded that these mechanisms all together promote oxidative damage induced by hyperandrogenism and consecutive CV damage confirmed by oxidative stress in CVE. However, the mentioned studies evaluated the effect of T in male rats, and our study was (to our knowledge) the first to investigate the effects of chronic T administration in a specific developmental window (prepubertal) to mimic PCOS on cardiodynamic and vascular functions in female rats. This is the first study which puts in focus on the hyperandrogenic milieu of PCOS in rats through the CV outcome regarding oxidative damage.

HR was not significantly altered in the PCOS group, although the values were lower than those of control. However, in the EV-induced PCOS rat model [56], reduction of the RR interval was demonstrated as a consequence of increased sympathetic activity. Different results could be attributed to different steroids used for PCOS induction. In women with PCOS, the basal heart rate was not altered [57], which was also observed in our rat study.

Regarding CF estimation, different CF parameters in PCOS patients compared to control describe the nonrestrictive diastolic function as well as increased LV stiffness [58]. These data were discussed as a consequence of hyperinsulinemia and confirmed by a positive correlation between EF and insulin levels. Coronary flow reserve has not changed between PCOS and control women [57] measured at the left anterior descending coronary artery. Although we did not measure such specific parameters, baseline coronary flow in PCOS rats had similar values in both groups, at all perfusion pressure values (40-120 mmHg).

The literature data demonstrated the relationship between BP levels and PCOS [59], even independent of obesity [60] in young patients. In our research, after 5 weeks of testosterone treatment, SBP showed borderline elevation compared to control values, but the registered PCOS values were not hypertensive. These data are in line with the study which demonstrated that ambulatory BP values in PCOS patients tend to increase [9]. Steroid-induced PCOS in rats resulted in an increase in SBP after 5 weeks of treatment [12]. In our study, MAP values were significantly higher in PCOS rats, which was observed by others as well [12, 61]. These data could be predictive for hypertension development in older age [62]. The exact mechanism which connects BP and PCOS has not been unraveled yet. There are proofs that androgens could modulate BP levels in genetically hypertensive species, such as SHR, since administration of the androgen antagonist resulted in a reduction of BP in young female SHR [63]. Moreover, it is known that female SHR had higher T levels, and T level in male SHR showed a marked rise in concentration from 8 weeks of age, the time when blood pressure showed higher values [64]. On the other hand, the endothelial-dependent vasodilatory effects of estrogen in females were described in the rat aorta [65], mechanistically by increasing the expression of eNOS. The unbalance between estrogen and T in female rats, as observed in our study, could be, at least partially, responsible for the lack of vasodilatory effects of estrogen-regulated vasoactive substances in PCOS rats, like NO. In favor of interpreting our results, the estrogen-protective effects on the CV profile proved useful via decreasing the endothelium-derived generation of O_2_^−^ and increasing endothelium-derived NO bioactivity [66], even without change in eNOS gene expression. Lower levels of nitrites in the systemic circulation of PCOS rats in our study could be at least related to the absence of NO-dependent vasodilatation and the tendency to develop higher BP values. The controversial evidence regarding T and oxidative stress could be found in literature: a large quantity of prooxidative T features and deleterious actions in the CV system were described [67]. Although the precise relationship between androgens and hypertension in women remains to be unraveled in further investigations, it is obvious that the effect of long-lasting administration of androgens in the female population leaves marked traces in terms of high BP levels even after months of treatment cessation [68]. Besides higher T level, increased sympathetic activity was suggested as one of the common features between PCOS and hypertension, even because resection of the ovarian superior nerve in rats led to diminishing PCOS characteristics in a rat model [69]. Increased sympathetic activity, which was described in PCOS rats, was also present in male SHR, and oxidative stress could have mediated observed changes. The recent data from our laboratory showed that levels of H_2_O_2_ were elevated and NO_2_^−^ reduced in male SHR [70], in addition to decreased (but not significant) levels of SOD and GSH. This similarity could be attributed to higher androgen levels in female PCOS rats. On the other hand, increased O_2_^−^ observed in the PCOS group in CVE, without change in nitrite levels, probably indicates a cardiotoxic effect of androgen in the heart by producing ROS. A decrease in H_2_O_2_ concentrations in CVE, particularly at higher perfusion pressures, observed in PCOS rats (Figure 4(b)), could be due to elimination by catalase activity in the heart, which was elevated (but not significant) in heart tissue. The increased O_2_^−^ levels in SHR arterioles could be related to hypertension [71]. Moreover, in the kidney, testosterone enhances O_2_^−^ production in the macula densa [72], which could be potentially responsible for higher incidence of hypertension in males compared to females. The role of oxidative stress in hypertension is well established, indicating that the autocrine or paracrine effect of increased ROS or diminished antioxidant capacity could be associated with higher blood pressure values [73]. Moreover, results from our laboratory concerning oxidative stress parameters in CVE of male SHR (unpublished data) showed similar alterations of O_2_^−^ and H_2_O_2_, without alterations in TBARS and NO levels. This similarity of oxidative stress parameters in SHR with results of the present androgen-induced PCOS model led us to the conclusion that androgen-dependent redox disturbance could mediate the potential mechanism for altered CV patterns in terms of the (pre)hypertensive state in PCOS. This claim was supported by the fact that in a salt-induced hypertension model, there was no alteration in oxidative stress parameters in CVE [74]. Observed together, the changes in most toxic prooxidants, like O_2_^−^ and H_2_O_2_, could be attributed to higher T levels.

## 5. Conclusion

In summary, PCOS rats showed increased contractile and enhanced lusitropic properties of LV and a tendency to increase arterial SBP. The morphometric heart tissue analysis showed hypertrophic characteristics in hyperandrogenic female rats. Rats with PCOS showed changes in prooxidative and antioxidative parameters in the systemic circulation, CVE, and heart tissue, which could have contributed to the modification of CV properties. Findings of the present study may help in revealing mechanisms of CV manifestation in PCOS and could be an excellent basis for further clinical investigations.

## Figures and Tables

**Table 1:** Values of the heart and final body weight measurements, as well as relative heart weight, expressed as their ratio (calculated by the formula HW/BW∗100). Data were presented as means ± SEM (*n* = 6 rats per group).

	Heart weight (HW, g)	Final body weight (FBW, g)	HW/FBW ratio
CTRL	1.03 ± 0.02	230 ± 4.47	0.45 ± 0.01
PCOS	1.21 ± 0.03	246 ± 2.08	0.49 ± 0.01
Significance	*p* < 0.01	*p* < 0.05	*p* < 0.05

## Data Availability

The data used to support the findings of this study are available from the corresponding author upon request.
